# A superiority of viral load over CD4 cell count when predicting mortality in HIV patients on therapy

**DOI:** 10.1186/s12879-019-3781-1

**Published:** 2019-02-15

**Authors:** Claris Shoko, Delson Chikobvu

**Affiliations:** Department of Mathematical Statistics and Actuarial Sciences, University of the Free State, Box 339, Bloemfontein, 9300 South Africa

**Keywords:** CD4 cell count, Viral load, HIV/AIDS progression, Multistate modelling

## Abstract

**Background:**

CD4 cell count has been identified to be an essential component in monitoring HIV treatment outcome. However, CD4 cell count monitoring sometimes fails to predict virological failure resulting in unnecessary switch of treatment lines which causes drug resistance and limitations of treatment options. This study assesses the use of both viral load (HIV RNA) and CD4 cell count in the monitoring of HIV/AIDS progression.

**Methods:**

Time-homogeneous Markov models were fitted, one on CD4 cell count monitoring and the other on HIV RNA monitoring. Effects of covariates; gender, age, CD4 baseline, HIV RNA baseline and adherence to treatment were assessed for each of the fitted models. Assessment of the fitted models was done using prevalence plots and the likelihood ratio tests. The analysis was done using the “msm” package in R.

**Results:**

Results from the analysis show that viral load monitoring predicts deaths of HIV/AIDS patients better than CD4 cell count monitoring. Assessment of the fitted models shows that viral load monitoring is a better predictor of HIV/AIDS progression than CD4 cell count.

**Conclusion:**

From this study one can conclude that although patients take more time to achieve a normal CD4 cell count and less time to achieve an undetectable viral load, once the CD4 cell count is normal, mortality risks are reduced. Therefore, both viral load monitoring and CD4 count monitoring can be used to provide useful information which can be used to improve life expectance of patients living with HIV. However, viral load monitoring is a better predictor of HIV/AIDS progression than CD4 cell count and hence viral load is deemed superior.

**Electronic supplementary material:**

The online version of this article (10.1186/s12879-019-3781-1) contains supplementary material, which is available to authorized users.

## Background

CD4 cell count and viral load (HIV RNA) count are the laboratory markers that are regularly used for HIV/AIDS patient management in addition to predicting disease progression and/or treatment outcomes [1]. The target of ART is to suppress the levels of HIV RNA in the plasma as this leads to increase in CD4 cell count and consequently reduces the risks of clinical events and the development of drug resistance [2].

CD4 cell count has been deemed an essential component of HIV treatment and care programmes since HIV was identified as a disease compromising the immune system [3]. Although the World Health Organisation (WHO) has recommended a shift to HIV RNA in monitoring ART, it continues to emphasise CD4 cell count’s importance in evaluating disease status at baseline and appropriate care for patients with advanced stages of HIV progression [3].

HIV RNA is most useful in measuring effectiveness of ART after initiation. Some researchers argue that lack of HIV RNA monitoring leads to delayed and unnecessary switches to second line therapy resulting in development of resistance to treatment and limitations to treatment options [4]. Other researchers argue that HIV RNA appears to be the best predictor of long-term clinical outcome whereas CD4 cell count predicts clinical progression and survival in the shorter term [5]. Brennan and others, in their research to determine the interplay between CD4 cell count and viral load, further argued that long-term virological suppression plays an important role in ensuring the recovery of CD4 cell count to levels that reduce the risk of opportunistic infection and increase life expectancy [6].

In the year 2000 there were uncertainties regarding the use of either CD4 cell count or viral load markers in controlled trials [5]. Thereafter, attempts have been made by different researchers to try and address these uncertainties. Some of these studies used Cox proportional hazard models and Kaplan Meier curves [2, 7]. Another study to establish the interplay between CD4 cell count, viral load suppression and duration of ART on mortality in a resource limited setting was carried out using log-linear model with Poisson distribution [8, 6]. However, results from these studies were contradictory. Some studies show that CD4 cell count monitoring is the best for predicting HIV/AIDS progression [4, 7, 8] and other studies show that viral load monitoring is the best predictor [1].

When HIV RNA tests are done, the results cannot be reliable due to missing data as a result of limiting costs [1]. Researchers then resort to use of computer simulated data [4]. In this study, longitudinal data collected from a Wellness clinic in Bela Bela, South Africa on viral load count monitoring and CD4 cell count monitoring, is analysed. A stochastic Markov approach to multistate modelling is used in the analysis. The objective of the study is to investigate and compare the use of either CD4 cell count or viral load markers in controlled trials. The aim is to determine whether CD4 cell count or viral load count can be used to model HIV/AIDS progression. Multistate modelling is a powerful tool for studying chronic diseases and in estimating factors associated with transitions between each stage of progression [9].

In the section that follows, methods used in the analysis of the data are explained. This is followed by section 3 on results and discussions. Lastly in section 4, conclusions of the findings are highlighted.

## Methods

### Data description

The data used in this study was obtained as secondary data from the University of Venda in South Africa. The names of participants were removed from the data set and as such the Ethics Committee of the University of Venda approved the usage of the data in 2013 (Additional file 1).

This study includes a selection of 320 HIV patients on anti-retroviral therapy (ART) who fulfilled the entry criteria from a longitudinal cohort of 1092 HIV-infected patients followed at a Wellness clinic in Bela Bela, South Africa, from year 2005 to year 2009. Patients were eligible for inclusion if they had a routinely reported viral load count and if they were 15 years and older. Upon initiation of treatment therapy, follow up was done in the first 3 months of treatment initiation and 6 months intervals thereafter. From these patients, 224 were females and 96 were males. The ages of the patients ranged from 15 years to 77 years and the children born to HIV+ patients were not included in the study. At baseline age, the data set had a first quartile of 32 years, a median of 39 years, a mean of 39.44 years and a third quartile of 47 years. One hundred seventy-two patients were aged 45 and below, and 72 were over 45 years of age. The viral load count at baseline of the patients ranged from 45 to 818,600 copies/mL with a mean viral load of 138,208 copies/mL, a first quartile of 21,334, a median of 67,995 and a third quartile of 201,445 copies/mL. From these patients, 267 had a viral load baseline above 10,000 copies/mL and 49 had a viral load baseline below 10,000 copies/mL. The CD4 baseline of the patients ranged from 16 to 1202 cells/mm^3^. The mean CD4 baseline was 156 cells/mm^3^, first quartile of 38 cells/mm^3^, median of 116 cells/mm^3^ and a third quartile of 206 cells/mm^3^. Approximately 70% of these patients had a CD4 baseline below 200 cells/mm^3^ (AIDS defining stage).

For each and every visit time, blood samples were obtained for each patient and stored frozen until assayed. Plasma HIV RNA was measured using an amplicator HIV-1 monitor assay kit which has a lower limit of sensitivity of 50 copies/mL.

At *t* = 0 the ART regimens that were mostly administered to patients were the triple combination therapy, D4T-3TC-EFV (208 patients) and D4T-3TC-NVP (92 patients). D4T and 3TC represent Stavudine and Lamivudine respectively which fall under nucleoside reverse transcriptase inhibitors (NRTI) class. EFV and NVP stand for Efavirenz and Nevirapine respectively and are from the non-nucleoside reverse transcriptase inhibitors (NNRTI) class. In patients who showed some signs of non-adherence, D4T was substituted with AZT (Zidovudine). A switch from D4T-3TC-EFV to AZT-3TC-EFV was most common rising from 10 patients in the first 6 months to 92 patients in 30 months (2 and half years). During the same period the number of patients who switched from D4T-3TC-NVP to AZT-3TC-NVP rose from 6 to 45. After 1 year of treatment uptake, one patient was introduced to FTC-TDF-EFV and after three and half years the frequency increased to 10 patients. Another combination of FTC-TDF-NVP was also introduced to 3 patients after 2 years. The number for this combination rose to 7 after 3 years. The drug regimens that were mostly administered during the first three and half years are summarised in the table below; (Table 1)

**Table 1:** Treatment regimen administered to the patients i the first 3.5 years of treatment follow-up

Drug/t	0	0. 25	0.5	1	1.5	2	2.5	3	3.5
1	208	191	165	140	94	44	18	5	3
2	92	73	70	62	35	23	7	1	0
3	2	3	10	20	50	77	92	88	60
4	3	6	6	14	35	36	45	35	31
5	0	0	0	1	1	3	8	10	10
6	0	0	0	0	0	3	5	7	3
7	2	2	1	2	5	4	2	2	1

KEY: 1:-D4T-3TC-EFV, 2:-D4T-3TC-NVP, 3:-AZT-3TC-EFV, 4:-AZT-3TC-NVP, 5:-FTC-TDF-EFV, 6:-FTC-TDF-NVP, 7:-D4T-3TC-LPV/r, *t* represents time in years post treatment commencement

During the course of the study, HIV/AIDS progression was assessed based on CD4 cell count monitoring, viral load count monitoring and also signs of non-adherence to treatment were noted. Patients who had problems in adherence to treatment were those patients who were intolerant to the treatment combination and those who failed to reach viral suppression. Change of treatment line was based on treatment failure, toxicity, patient intolerance to the combination therapy or inability of the patient to adhere to treatment and viral rebound. From these patients, 36 showed some signs of non-adherence to treatment. In this study, viral load below 50 copies/mL is defined as undetectable viral load and the progression of HIV/AIDS is defined either by change in viral load count level or change in CD4 count level. The viral load count levels are divided into 5 transient states and the sixth state being the absorbing state, death. The CD4 count levels are divided into 4 transient states and the fifth state is the death state. The viral load states, CD4 states as well as factors that are likely to determine change in viral load/CD4 states are defined in the next sub-section.

### Variable coding

For this study, variables are coded as follows:

A. *Categorical variables*

$$ \mathrm{Age}=\kern0.5em \left\{\begin{array}{c}1,\le 45\  years\\ {}0,>45\  years\end{array}\right.,\mathrm{Non}\hbox{-} \mathrm{adherence}\ \left(\mathrm{NA}\right)=\left\{\begin{array}{c}1, Yes\\ {}0, No\end{array}\right., $$

$$ \mathrm{CD}4\ \mathrm{baseline}\ \left(\mathrm{CD}4\mathrm{BL}\right)=\left\{\begin{array}{c}1,\le 200\ \mathrm{cells}/{\mathrm{mm}}^3\\ {}0,>200\ \mathrm{cels}/{\mathrm{mm}}^3\end{array},\mathrm{Gender}=\right.\left\{\begin{array}{c}1, male\\ {}0, female\end{array}\right., $$

$$ \mathrm{viral}\ \mathrm{load}\ \mathrm{baseline}\ \left(\mathrm{VLBL}\right)=\left\{\begin{array}{c}1,>10\ 000\  copies/ mL\\ {}0,\le 10\ 000\  copies/ mL\end{array},\right. $$

B. *Time-dependent variables*

$$ \mathrm{Viral}\ \mathrm{load}\ \mathrm{levels}\ \left(X\;(t)\right)=\left\{\begin{array}{c}\mathbf{1}; VL<50\\ {}\mathbf{2};50\le VL<10\ 000\\ {}\mathbf{3};10\ 000\le VL<100\ 000\\ {}\mathbf{4};100\ 000\le VL<500\ 000\\ {}\mathbf{5}; VL\ge 500\ 000\\ {}\mathbf{6}; Dead.\end{array}\right. $$

$$ \mathrm{CD}4\ \mathrm{cell}\ \mathrm{count}\ \mathrm{levels}\ \left(\;X(t)\right)=\left\{\begin{array}{c}\mathbf{1}; CD4>800\\ {}\mathbf{2};500< CD4\le 800\\ {}\mathbf{3};350< CD4\le 500\\ {}\mathbf{4}; CD4<350\\ {}5; Death\end{array}\right. $$

The effects of the categorical variables on the time-dependent variables is assessed using the Markov models:

$$ {q}_{ij(CD4)}={q}_{ij(CD4)}^{(0)}\exp \left({\upbeta}_{ij}^{(Age)}{Age}_h+{\beta}_{ij}^{(Gender)}{Gender}_h+{\beta}_{ij}^{\left( CD4 BL\right)} CD4{BL}_h+{\beta}_{ij}^{(VLBL)}{VLBL}_h+{\beta}_{ij}^{(NA)}{NA}_h\right) $$

and

$$ {\displaystyle \begin{array}{l}{q}_{ij(VL)}={q}_{ij(VL)}^{(0)}\exp \left({\beta}_{ij}^{(Age)}{Age}_h+{\beta}_{ij}^{(Gender)}{Gender}_h+{\beta}_{ij}^{\left( CD4 BL\right)} CD4{BL}_h+{\beta}_{ij}^{\left( VL BL\right)}{VLBL}_h+{\beta}_{ij}^{(NA)}{NA}_h\right)\\ {}\end{array}} $$

for CD4 cell count levels and viral load levels respectively. ${q}_{ij(CD4)}^{(0)}$ and ${q}_{ij(VL)}^{(0)}$ are the baseline transition intensities for CD4 cell count states and viral load states respectively. *β*_*ij*_ is the log-linear effects of the mentioned covariate on the baseline transition intensities ${q}_{ij}^{(0)}$.

## Results

**Fig. 1:**
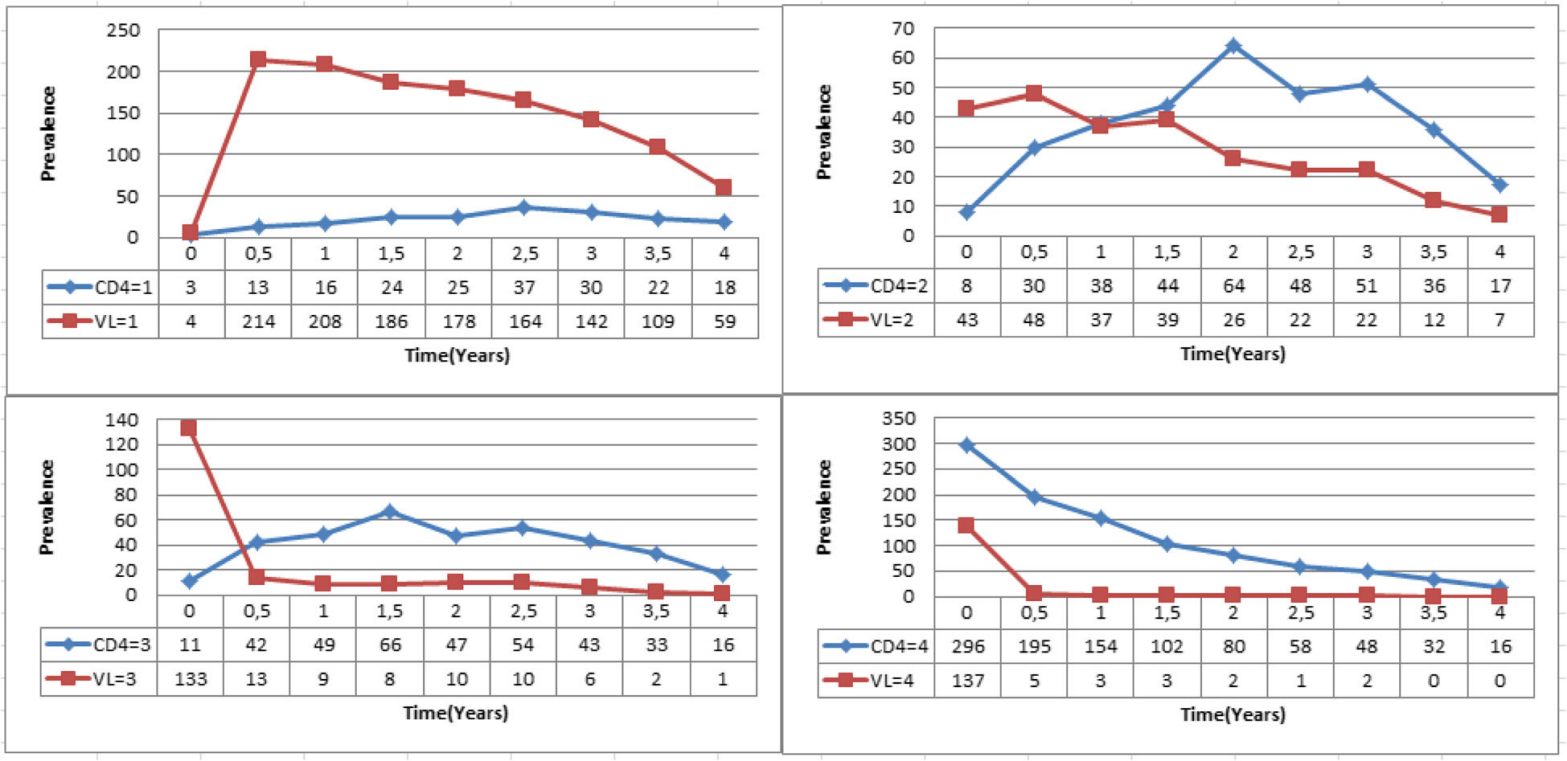
Comparison of CD4 and Viral load prevalence 4 years post commencement of therapy(Original). Legend: CD4 stages:1:- CD4 > 800, **2:-**  500 < CD4 ≤ 800, **3:-** 350 < CD4 ≤ 500;4:- CD4 < 350; Viral load states:1:- VL < 50, **2:-**  50 ≤ VL < 10 000, **3:- ** 10 000 ≤ VL < 100 000, **4:-**  VL ≥ 100 000

The observed prevalence for each of the variables CD4 cell count and viral load count were computed in R using the “msm” package for multistate modelling. The observed prevalence are calculated for each CD4 cell count state and viral load count state. This is done from initiation of treatment (t = 0 years) to time t = 4 years. The comparison is based on the transient states based on either CD4 cell count or viral load levels. However, since viral load states are more than CD4 count states, viral load state 4 and state 5 are combined so that we have an equal number of transient states for both variables. The results are shown in Fig. 1 below.

Results from Fig. 1 above show an increase in the number of patients who had their viral load suppressed/undetectable in the first 6 months of treatment uptake. The plotted variables are shown at the bottom of each graph. From 6 months onwards, the number individuals with suppressed viral load started to decrease. This could be caused by loss of viral suppression or deaths. The number of patients with CD4 cell count above 800 (CD4 state = 1) increased slowly with time. In 2014, Maartens and others also indicate that within 3 months of ART, the plasma viral load decreases to concentrations below the lower limit of detection of available commercial assays in most people [10]. The lower limit for this particular study is 50 copies/mL.

Upon initiation of treatment, the majority of the patients had a viral load state equal to 3, which is associated with viral load count of between 10,000 and 100,000 copies/mL. After 6 months of ART the number of patients in this category dropped from 133 to 13 and continued to decrease throughout the whole period. The highest number of patients was in the CD4 cell count category 4, which is defined by a CD4 cell count below 350 cells/mm^3^. The number of patients in this state continued to decrease throughout the whole period but at a slower rate than that of viral load count levels.

### Effects of CD4 levels on viral load count transition intensities

In this sub-section, we analyse the effects of CD4 cell count levels on transition intensities defined by viral load as defined by the equation:

$$ {\alpha}_{ij(VL)}={\alpha}_{ij}^0\exp \left({\beta}_{ij}\times CD{4}_k\right) $$

where *α*_*ij*(*VL*)_ is the transition intensity matrix for *i* = 1, … , 5 transient states defined by viral load levels in the plasma cells and *j* = 1, … , 6, *β*_*ij*_ is the log-linear effect of CD4 cell count level on the transition intensity *α*_*ij*(*VL*)_ and *k* = 1, … , 4 defines the different levels of CD4 cell count. For this model, transition from *i* to *j* where *i* > *j* is defined as viral load count suppression and if *i* < *j,* it is defined as viral rebound*.* The values of *k* define the patient’s immunology such that large values of *k* are associated with immune deterioration and smaller values of *k* are associated with immune recovery. ${\alpha}_{ij}^0$ is the baseline transition intensity from *i* to *j .* The results of the transitions are shown in Table 2 below.

**Table 2:** Effects of changes in CD4 cell count levels on viral load transition intensities

	Baseline	Log-linear	Hazard	CD4Level			
*α*_*ij*_	${\alpha}_{ij}^0$	*β*_*ij*_		*k* = 1	*k* = 2	*k* = 3	*k* = 4
*α*_12_	0.4679	0. 2451	1. 2778	0. 2685	0.3429	0.4380	0.5595
*α*_16_	0.0170	0.0732	1.0760	0.0144	0.0155	0.0167	0.0179
*α*_21_	3.1857	0.0612	1.0631	2.7644	2.9426	3.1325	3.3345
*α*_23_	5.6586	0.8919	2.4397	0.7877	1.8778	4.4768	10.6725
*α*_26_	0.1388	1.5639	4.7776	0.0044	0.0201	0.0921	0.4222
*α*_32_	30.4528	0.8684	2.3831	4.4659	10.4049	24. 2421	56.4807
*α*_34_	3.1488	−0.0002	0.9998	3.1766	3.1644	3.1521	3.1399
*α*_36_	0.0072	−0.1439	0.8660	0.0126	0.0099	0.0077	0.0060
*α*_43_	16.9641	0.5376	1.7119	5.0556	8.6181	14.6914	25.0443
*α*_45_	2. 2611	1. 2750	3.5789	0.1182	0.4339	1.5924	5.8438
*α*_46_	0.0096	−1.7277	0.1777	0.6964	0.1056	0.0160	0.0024
*α*_54_	6.5317	1.0211	2.7762	0.6131	1.7387	4.9311	13.9850
*α*_56_	0.0451	−2.5302	0.0796	23.7348	1.5009	0.0949	0.0060
-2xLL	2665. 285						

*α*_*ij*_:- transition intensities, ${\alpha}_{ij}^0$ baseline transition intensities, *β*_*ij*_:- log-linear effects, *Hazard:-* hazard ratios, *CD4Level:-* CD4 cell count transient states, *2xLL:-* likelihood ratio test

Results from Table 2 above show that the rates of viral suppression are higher than the rates of viral rebound for HIV+ patients in state 3 (viral load ranging from 10,000 to below 100,000 copies/mL), state 4 (viral load level ranging from 100,000 to below 500,000 copies/mL) and state 5 (viral load level above 500,000 copies/mL). If a patient is in a viral load level suppressed to state 2 (from 50 to below 10,000 copies/mL), the rates of viral rebound to state 3 are higher than the rates further viral load suppression to state 1.

For the viral rebound from state 1 (undetectable viral load) to state 2, the log-linear effect of CD4 count level is positive. This indicates that viral rebound from the undetectable level increases as the immune system deteriorates. The increase in transition intensities from 0.2685 at *k* = 1 to 0.5595 at *k* = 4 confirms the increase in viral load as the immune system deteriorates. Although the log-linear effects of CD4 cell count levels on viral rebound and viral suppression from state 2 are both positive, the effect on viral rebound is higher and this also increases as the immune system deterioration. This means that a patient can reach a suppressed viral load yet the immune system is still compromised (CD4 cell count still low).

When the viral load level is 3 and above (viral load of 10,000 copies/mL and above) mortality rates decrease with immune deterioration. Mortality rates increase with immune deterioration for viral load count levels is below 10,000 copies/mL. This means that during the early phases of treatment uptake, when the viral load levels are high and the CD4 count levels are still low, there are low of transitions death rates. Deaths are mainly caused by viral rebounds due to a compromised immune system.

### Effects of viral load levels on CD4 cell count transition intensities

In this sub-section we analyse the effects of viral load levels on transition intensities defined by CD4 cell count as defined by the equation:

$$ {\alpha}_{ij(CD4)}={\alpha}_{ij}^0\exp \left({\beta}_{ij}\times {VL}_k\right) $$

where *α*_*ij*(*CD*4)_ is the transition intensity matrix for *i* = 1, … , 4 transient states defined by CD4 cell count levels and *j* = 1, … , 5, *β*_*ij*_ is the log-linear effect of viral load count level on the transition intensity *α*_*ij*(*CD*4)_ and *k* = 1, … , 5 defines the different levels of viral load. For this model transition where *i* > *j* is defined as immune recovery and if *i* < *j*, it is defined as immune deterioration*.* The values of *k* define the patient’s virology such that large values of *k* are associated with high level of viral load and smaller values of *k* are associated with suppressed viral load. The results are shown in Table 3 below.

**Table 3:** Effects of changes in viral load levels on CD4 cell count transition intensities

	Baseline	Log-linear	Hazard	Viral load levels				
*i;j*	${\alpha}_{ij}^0$	*β*_*ij*_		1	2	3	4	5
1;2	0. 2901	−1.4148	0. 2430	0.7161	0.1740	0.0423	0.0103	0.0025
1;5	0.0240	1. 2788	3.5922	0.0106	0.0381	0.1369	0.4918	1.7665
2;1	0.6124	0.02164	1.0268	0.6021	0.6182	0.6348	0.6518	0.6692
2;3	0.8429	0. 2122	1. 2364	0.7361	0.9101	1.1252	1.3911	1.7199
2;5	0.0044	1.5472	4.6985	0.0016	0.0076	0.0358	0.1684	0.7912
3;2	1.3971	0. 2010	1. 2226	1.2287	1.5023	1.8367	2.2456	2.7454
3;4	0.7200	0. 2729	1.3138	0.6048	0.7946	1.0440	1.3716	1.8019
3;5	0.1276	1.0505	2.8591	0.0652	0.1865	0.5331	1.5241	4.3576
4;3	0.7432	0.0231	1.0233	0.7323	0.7494	0.7669	0.7847	0.8030
4;5	0.0567	0.5734	1.7743	0.0393	0.0697	0.1237	0.2195	0.3894
-2xLL	3308.126							

*α*_*ij*_:- transition intensities from state *i* to state *j*, ${\alpha}_{ij}^0$ baseline transition intensities, *β*_*ij*_:- log-linear effects, *Hazard:-* hazard ratios, -2xLL*:-* likelihood ratio test

The results from Table 3 show that the rates of immune deterioration are lower than the rates of immune recovery when a patient’s CD4 cell count is 500 cells/mm^3^ and below (state 3 and state 4). When the CD4 cell count levels are above 500 cells/mm^3^ (states 1 and 2) rates of immune deterioration are higher than rates of immune recovery. This is an indication that upon reaching the safe immunological levels, there are certain factors that compromise the immune system. There is need to further investigate the cause.

The negative log-linear effect of viral load levels on the transition from state 1 (CD4 count above 800) to state 2 (CD4 count more than 500 but less or equal to 800 cells/mm^3^) indicates a reduction in immune deterioration from state 1 to state 2 as the levels of viral load in the plasma increase. Mortality rates from all the states increase as the viral load levels increase. The highest transitions to death are recorded for patients with viral load levels above 500,000 copies/mL (state 5).

### Effects of covariates on CD4 cell count and viral load levels

Effects of covariates; Age, Gender, VL baseline (VLBL), CD4 baseline (CD4BL), Non-adherence to treatment (NA) on HIV/AIDS progression defined by the time-dependent variables CD4 levels or viral load levels is assessed in this section. The models for the effects of covariates on transition intensities defined by CD4 cell count and viral load are:

$$ {q}_{ij(CD4)}={q}_{ij(CD4)}^{(0)}\exp \left({\upbeta}_{ij}^{(Age)}{Age}_h+{\beta}_{ij}^{(Gender)}{Gender}_h+{\beta}_{ij}^{\left( CD4 BL\right)} CD4{BL}_h+{\beta}_{ij}^{(VLBL)}{VLBL}_h+{\beta}_{ij}^{(NA)}{NA}_h\right) $$

and

$$ {\displaystyle \begin{array}{l}{q}_{ij(VL)}={q}_{ij(VL)}^{(0)}\exp \left({\beta}_{ij}^{(Age)}{Age}_h+{\beta}_{ij}^{(Gender)}{Gender}_h+{\beta}_{ij}^{\left( CD4 BL\right)} CD4{BL}_h+{\beta}_{ij}^{\left( VL BL\right)}{VLBL}_h+{\beta}_{ij}^{(NA)}{NA}_h\right)\\ {}\end{array}} $$

respectively. *β*_*ij*_ is the log-linear effects of the mentioned covariate on the baseline transition intensities ${q}_{ij}^{(0)}$.

The results show no gender effect on the progression of HIV based on viral load levels. This means that change in viral load levels is uniform for both males and females. However, given the time-dependent variable CD4 cell count, the effects of gender is quite significant. Thus, in Table 4 below, when the CD4 cell count is below 350 cells/mm^3^, males have lower chances of immune recovery than females. The effects of gender are only indicated for CD4 cell count levels. Similar results for viral load levels are not presented since they are not significant.

**Table 4:** Log-linear effects of age, viral load baseline, CD4 baseline, gender and non-adherence on baseline transition intensities for CD4 and viral load stages

			Log-linear effects (*β*_*ij*_)								
	Baseline (*q*_*ij0*_)		Age		VLBL		CD4BL		Gender	Non-adherence	
*i;j*	CD4	VL	CD4	VL	CD4	VL	CD4	VL	CD4	CD4	VL
1;2	0.7373	0.4957	−1.3266	−0.1479	−0.1436	0.1153	−0.4644	− 0.0973	− 0.2887	− 0.2319	0.2189
1;death	0.0003	0.0001	− 0.7467	4.4953	1.1724	3.4155	0.9160	3.5811	0.5660	−0.0148	4.4320
2;1	0.5699	4.025	0.3444	−0.4369	−0.1826	− 0.3702	−0.3360	0.3262	0.1063	0.7056	−1.3054
2;3	0.7515	6.068	−0.0925	0.4862	0.4300	2.4328	−0.0483	−2.8265	0.8865	0.9685	3.2746
2;death	0.0026	0.0058	4.3919	−1.5407	4.1370	3.4727	−0.0313	5.2590	1.8332	−2.2185	−5.0841
3;2	1.2831	62.87	0.2862	0.0611	−0.1617	0.8531	−0.5877	−2.9784	0.1138	0.1224	1.9255
3;4	0.7053	0.2084	0.0226	5.5325	−0.1768	1.1270	−0.4604	5.7147	−0.5035	0.6828	−0.2225
3;death	0.0001	0.0008	3.0206	−0.2825	2.0134	−0.3685	2.2871	0.1540	−3.9785	5.1871	−1.5359
4;3	0.7923	40.77	0.0223	0.4827	0.3822	−2.6884	−1.4319	0.8024	−0.5364	−0.3456	−0.7337
4;5	0.0005	0.5795	−2.0121	1.0819	3.6009	0.7886	3.3325	−2.2219	−5.8879	−4.0417	4.8056
4;6		0.0019		0.0607		−0.5510		−0.4202			−0.7981
5;4		100.5		−5.0660		−1.1924		2.1054			1.0696
5;6		0.0398		0.2639		1.8205		−3.4286			−2.0918
−2xLL	2595.89	1767.02									

Age = 1 if ≤45 years and 0 otherwise; *VLBL:-* viral load baseline =1 if >10000copies/mL and 0 otherwise, *CD4BL:-* CD4 baseline = 1 if ≤200 cells/mm^3^ and 0 otherwise; Gender = 1 if “male” and 0 if “female”; Non-adherence =1 if “yes” and 0 if “no”; −2xLL:-  likelihood ratio test

Results from Table 4 above show that for patients in the disease state 2, defined either by CD4 cell count levels or viral load levels, the rates of disease progression to state 3 are higher than the rates of recovery from state 2 to state 1. However, the rate of viral rebound is higher than the rate of immune deterioration for patients in state 2.

The results also show a reduction in viral load suppression from state 2 to state 1 and an increased viral rebound from state 2 to state 3 for patients who are 45 years and below compared to those patients over 45 years. The opposite is true for changes in CD4 cell count level. These patients, 45 years and below, show an increased immune recovery from state 2 to state 1 and a reduced immune suppression from state 2 to state 3. Although young patients experience some challenges in viral load suppression, they have higher chances of cell regeneration than their older counterparts.

Patients who initiated treatment with a viral load baseline above 10,000 copies/mL experience an increase in viral rebound and also an increase in immune deterioration from state 2 to state 3 and a reduced viral suppression and immune recovery from state 2 to state 1. However, it is interesting to note that if the patient’s CD4 cell count at treatment initiation is 200 cells/mm^3^ and below, there is increased viral load suppression from state 2 to state 1 and a decreased viral rebound from state 2 to state 3. This emphasises the need for initiation of treatment when the CD4 cell count is low to reduce the chances of reaction to treatment that are associated with long-term treatment uptake.

Patients with non-adherence to treatment have increased viral rebound from state 2 to state 3 and a decreased viral suppression from state 2 to state 1. Non-adherence to treatment causes an increased immune deterioration from state 2 to state 3. This also leads to an increased death rate from a CD4 state of 3. In general, given that a patient is non-adherent to treatment, there are increased rates of disease progression than recovery.

The results also show that deaths from viral load state 1(undetectable viral load) are higher for patients below the age group of 45 years than their older counterparts. However, for patients whose CD4 cell count has reached normal levels, transitions to death are lower in patients below 45 years than older patients. Deaths of patients below 45 years are prominent from a CD4 cell count states 2 and 3 compared to the older patients. For these patients in viral load levels 2 and 3 the opposite is true since lower transitions to death are observed from this data set when compared to the older patients. Thus, although HIV/AIDS patients take longer time to reach a normal CD4 cell count level than the time taken to reach a suppressed viral load count, once a normal CD4 cell count is reached mortality risks are reduced.

Patients who initially had a viral load baseline of more than 10,000 copies/mL experience higher transitions to death from almost all viral load states except state 4 and the highest transition to death are noted from state 2. For these individuals with initial viral load baseline above 10,000 copies/mL, the same trend is also notable from all the CD4 cell count states.

Patients with suppressed viral load who developed negative reaction to treatment (non-adherent to treatment) show the higher transitions to death compared to patients who did not develop any form of negative reaction to treatment.

In the next subsection prevalence plots for the two Markov models, one in which CD4 count is used as a marker of HIV/AIDS progression and the other one in which viral load count is used as the marker of the disease progression, are compared. The likelihood ratio test is also used assess the fitted models.

### Assessment of the fitted models

**Fig. 2:**
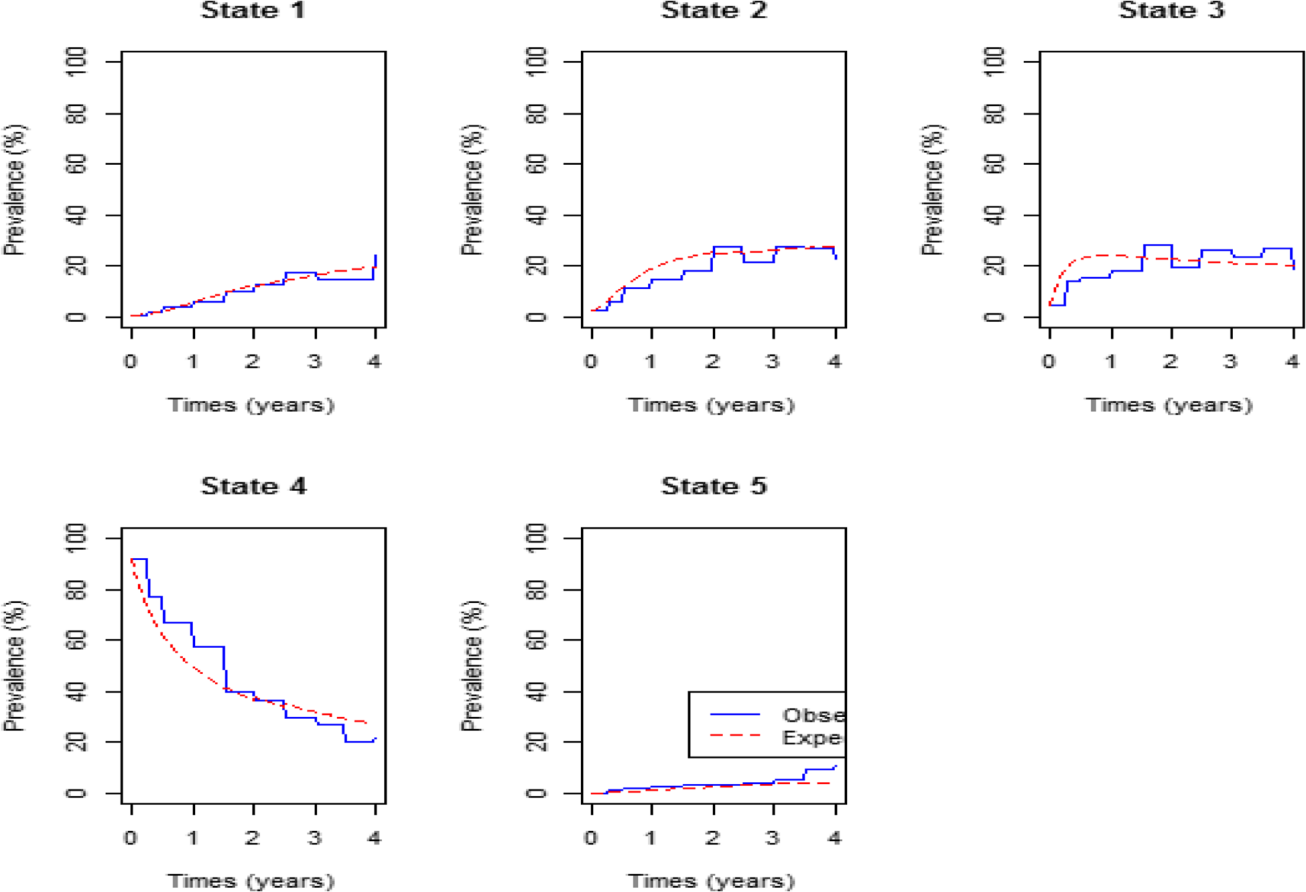
Percentage prevalence plot for the covariate on HIV/AIDS progression defined by CD4 cell count (Original). Legend: State:1= CD4 > 800, State2 = 500 < CD4 ≤ 800, State3=350 < CD4 ≤ 500; State4= CD4 < 350; State5 = death

Assessment of the fitted models is done by comparing the expected to the observed percentage prevalence. In Fig. 2 below, the comparison is based on CD4 cell count monitoring.

Figure 2 above show that at treatment initiation, more than 90% of the patients had a CD4 cell count below 200 cells/mm^3^ (state 4). As the time on treatment increases, the percentage prevalence for the patients in state 4 decreases exponentially to close to 20% after 4 years of treatment initiation. For CD4 states 1, 2 and 3, the percentage prevalence at initiation were close to 0% and increased exponentially to more than 20% in state 2 and 3 after 2 years of treatment and slightly above 10% for state 1. Thereafter the percentage prevalence for all the three states started to decrease, but at a slow rate. Death prevalence increases from 0% to approximately 10% in the first 4 years of treatment uptake.

**Fig. 3:**
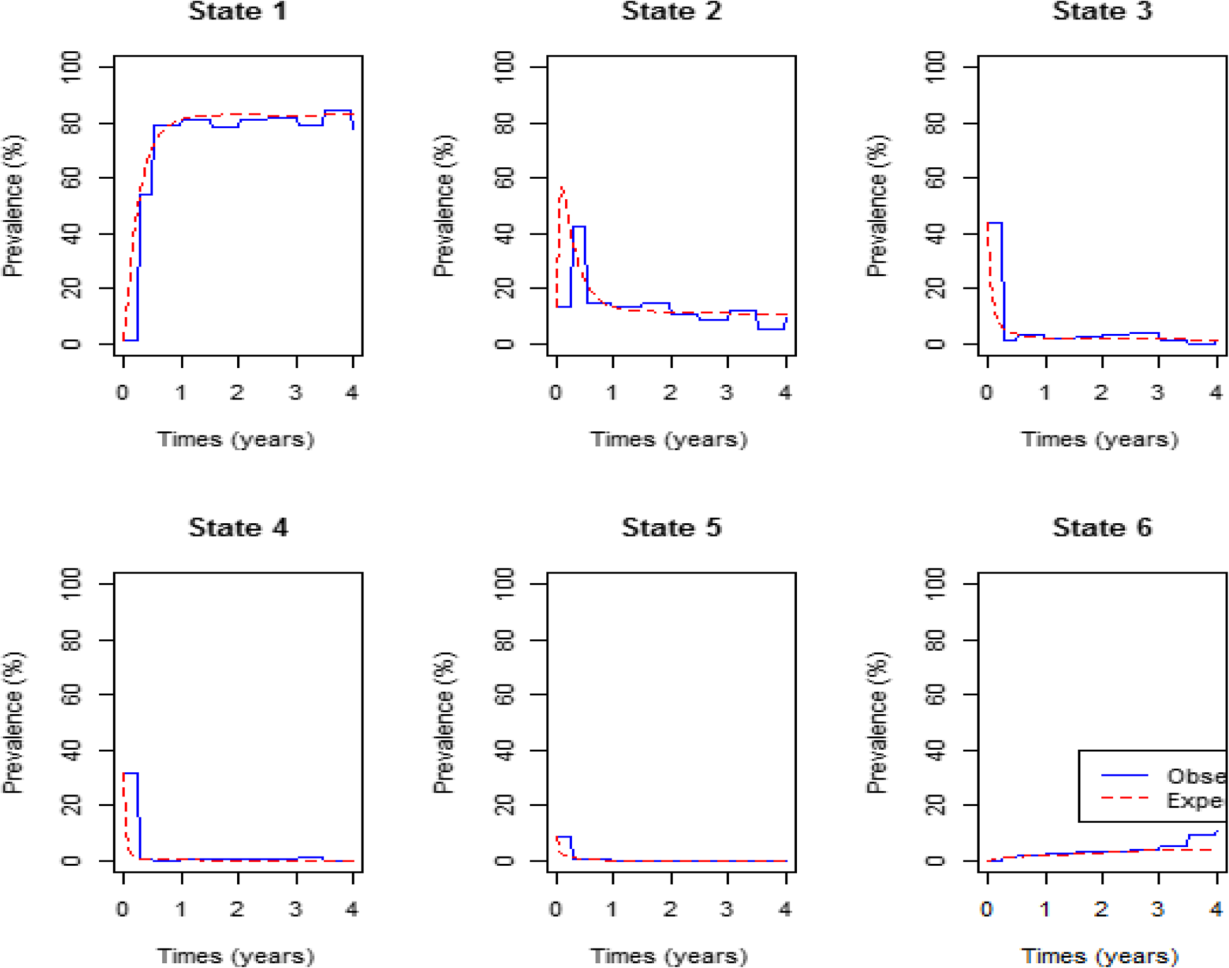
Percentage prevalence plot for the covariate on HIV/AIDS progression defined by Viral load (Original). Legend: state:1 = VL < 50, State2 = 50 ≤ VL < 10 000, State3 = 10 000 ≤ VL < 100 000, State4 = 100 000 ≤ VL < 500 000; State5 = VL≥ 500,000; State6 = death

In Fig. 3 below comparison of the expected percentage prevalence with the observed percentage prevalence is based on viral load levels.

Figure 3 shows that upon initiation of treatment more than 40% of the patients were in viral load state 3. This state had the highest percentage prevalence at start of therapy administration followed by state 4 which had close to 33%. Close to 0% of the patients had undetectable viral load levels (state 1) and this increased at a fast rate to approximately 80% after 1 year of treatment up take. After 1.5 years the percentage prevalence for state 1 became stable with a slight up and down trend. This could be due to viral rebound or deaths.

The model fitted for viral load states show a perfect fit for all the states. The model for CD4 states show a perfect fit only for state 1 percentage prevalence. States 2 and 3 overestimate the observed prevalence in the first 2 years of treatment. State 4 underestimates the observed in the first 1.5 years of treatment up take. The fitted model for CD4 states shown in Fig. 2, underestimates the observed death percentage prevalence slightly for the first 3.5 years and the margin become wider beyond 3.5 years. In Fig. 3, the model for viral load states show a perfect fit of the expected and observed death prevalence in the first 3.5 years but underestimates the observed death prevalence beyond. Thus, the fitted model for viral load states predicts mortality better than the model for CD4 states. This shows that progression of HIV/AIDS for patients on treatment is better explained by the changes in the viral load levels than the changes in the CD4 cell count levels.

A likelihood ratio test was also performed to compare HIV/AIDS progression based on CD4 cell count monitoring with progression based on viral load monitoring. The results yield a *p*-value of 10^{− 4}^ in favour of the Markov model based on the viral load monitoring. This again confirms that viral load monitoring is a better marker of HIV/AIDS progression than CD4 cell count. The results are shown below (Table 5).

**Table 5:** Log-ratio test for the superiority of viral load monitoring over CD4 cell monitoring

	-2 log LR	df	*p*-value
VL.cov1.msm	828.869	5	10^{−4}^

## Discussions

The major aim of this study was to assess and compare the use of the time-dependent variables; CD4 cell count and viral load level, in analysing HIV/AIDS progression on patients receiving antiretroviral therapy. Effects of covariates such as gender, age, CD4 baseline, viral load baseline, and adherence to treatment were also considered.

In this study, there are no gender effects on the progression of HIV/AIDS when viral load levels in the plasma are used as a surrogate marker. The results showed that gender effects are influenced by CD4 cell count. Previous findings by Dounelly et al. [11] demonstrate that women had non-significant lower viral loads than men and that the gender effects depended on CD4 cell count.

Patients below the age of 45 years had lower rates of viral load suppression to undetectable levels but they had faster rates of immune recovery compared to the older patients in the cohort (above 45 years of age). The results are corroborated by the findings from a study that was carried out in Tehran, Iran, which showed that mean CD4 cell count increments after initiation of antiretroviral therapy are lower on older patients (> = 50 years) [12]. Prior to the study by Hasib et al. [12], a study from Greece showed higher magnitudes of absolute CD4 cell count among patients 50 years and older [13]. The results reveal that, although HIV/AIDS patients generally take longer to reach a normal CD4 cell count compared to the time taken to reach an undetectable viral load, patients below the age of 45 years have reduced risks of mortality once the CD4 cell count is normal.

Overall, the results show that although both CD4 cell count and viral load are the surrogate markers of HIV progression, viral load is more powerful in monitoring progression of HIV/AIDS in patients on antiretroviral therapy than CD4 cell count. The models for viral load count with and without the inclusion of covariates give a better fit compared to the model for CD4 cell count (with and without the inclusion of covariates). This point coincides with WHO recommendations that advise the routine use of viral load monitoring as a routine procedure in the management of HIV infection. It goes further to recommend that in cases of treatment failure, where viral load testing is not routinely available, CD4 count can be used [14]. Deaths are well explained in the model for viral load monitoring than the model for CD4 cell count. This contradicts with findings from previous studies that CD4 cell count is a better predictor for HIV/AIDS progression than HIV RNA [7, 8, 15].

However, the 320 patients used in the study were selected on the basis that their viral load monitoring was routinely monitored throughout the study leading to selection bias. The limitation of this study was that although gender and age were considered in the analysis, the study disregarded the aspect of opportunistic infection.

## Conclusion

Although viral load monitoring in predicting HIV/AIDS progression gives the stakeholders a measure of understanding, control and motivation to adhere to treatment and enhances understanding of HIV infection [14]. It is recommended that both viral load monitoring and CD4 cell count monitoring be used since viral load determines the need for treatment change and CD4 cell count helps in monitoring the risk of opportunistic infection (OI) and treatment failure. From this study, one can also conclude that although patients take more time to achieve a normal CD4 cell count and less time to achieve an undetectable viral load, once the CD4 cell count is normal, mortality risks are reduced. Therefore, both viral load monitoring and CD4 count monitoring can be used to contribute information which can be used to significantly improve the life expectance of patients living with HIV.

## Additional file

Additional file 1: BMCID_Data. Data sheet containing all anonymised data used in this analysis. (XLSX 250 kb)

## Abbreviations

AIDS: Acquired Immune deficiency Syndrome
ART: Antiretroviral Therapy
CD4BL: CD4 Baseline
HIV: Human Immunodeficiency Virus
msm: Multistate modelling
NA: Non-adherence
OI: Opportunistic Infection
RNA: Ribonucleic Acid
VL: Viral load
VLBL: Viral Load Baseline
WHO: World Health Organisation
